# Development of a rating scale for maladaptive symptoms by maltreatment: Perspectives of attachment and dissociation

**DOI:** 10.1371/journal.pone.0298214

**Published:** 2024-02-14

**Authors:** Aika Horiuchi, Tomoko Nishimura, Masako Taniike, Masaya Tachibana

**Affiliations:** 1 Division of Developmental Neuroscience, United Graduate School of Child Development, Osaka University, Suita, Japan; 2 Research Center for Child Mental Development, Hamamatsu University School of Medicine, Hamamatsu, Japan; 3 Molecular Research Center for Children’s Mental Development, United Graduate School of Child Development, Osaka University, Suita, Japan; 4 Department of Pediatrics, Graduate School of Medicine, Osaka University, Suita, Osaka, Japan; Chiba Daigaku, JAPAN

## Abstract

Child abuse has been increasing in Japan. Abused children’s behavior may often be confused with neurodevelopmental disorders; therefore, specialized tools to identify these cases and specific care for maltreatment are crucial. This study aimed to develop an objective early screening scale for abuse-related maladaptive symptoms. To do this, two surveys were conducted. Survey 1 included 60 children attending public elementary schools, who had been admitted to orphanages due to abuse (maltreated group), and 154 children attending public elementary schools with no reported maltreatment (control group). In this survey, 40 existing scale items related to attachment behavior and dissociative symptoms were evaluated. Childcare staff and homeroom teachers evaluated children’s behaviors. Receiver operating characteristic (ROC) curves were drawn to determine optimal cut-off values. In Survey 2, 39 children in the maltreatment group and 186 children in the control group were subjected to confirmatory factor analysis to examine the new scale’s reliability and validity. Based on the results of an exploratory factor analysis, a two-factor, 20-item rating scale for maladaptive symptoms due to maltreatment (RS-MSM) was developed. The receiver operating characteristic curve indicated that cutoff values set in Survey 1 were appropriate for screening the general population and children in the clinical range. The results confirmed a two-factor structure with high reliability and convergent validity in the Survey 2 sample. Therefore, the developed RS-MSM scale is valid and will allow for easy screening of maltreated children at school.

## Introduction

In Japan, over 205,029 cases of abuse were reported in 2021. In 1990; however, the number of cases was 1,101 when the statistics were first published [1]. Simultaneously, the number of children who repeatedly exhibit problematic behaviors or difficulties in adjustment to school life without support in Japanese schools has increased [2].

Children who grow up in an environment of maltreatment and experience chronic trauma exhibit structural and functional changes in their brains [3] and some present with behavioral problems resembling neurodevelopmental disorders [4]. Alternatively, neurodevelopmental disorders, such as autism spectrum disorder (ASD) and attention-deficit/hyperactivity disorder (ADHD), are well-known risk factors for child maltreatment [5]. Thus, a proportion of children who are maltreated may present with neurodevelopmental disorders. Unlike children with developmental disorders without experiences of maltreatment, these children may exhibit symptoms of complex post-traumatic stress disorder (PTSD), such as dissociation and emotional dysregulation [6, 7].

Neurodevelopmental disorder-like behavioral problems in children who are maltreated are often considered developmental disabilities in school settings. However, reports indicate that support for developmental disorders alone is ineffective. These children are less likely to trust their caregivers due to a history of insufficient caregiving and more likely to interact in socially inappropriate ways [8].

Severer problems, such as maladjustment and truancy, can result from a lack of appropriate psychosocial support for such children, who are prone to trial behaviors due to attachment problems, and dissociation due to trauma reenactment. If the reason behind a child’s behavior problem is attributed to maltreatment, it needs early detection and addressal.

Various instruments have been developed to detect and assess maltreatment. The International Trauma Questionnaire (ITQ) [9] is available as a self-administered measure focusing on complex PTSD arising from maltreatment. Additionally, the psychometric properties of other self-assessment measures and scales which have been validated, including the Childhood Trauma Questionnaire (CTQ) [10], Maltreatment and Abuse Chronology of Exposure (MACE) scale [11], Child Abuse Potential Inventory (CAPI) [12], and the instrument of Identification of Parents At Risk for Child Abuse and Neglect (IPARAN) [13]. Children who are maltreated have ongoing psychological traumatic experiences. It is difficult to assess such experiences with a self-administered scale because these children may have dissociative symptoms, such as amnesia. Moreover, the risk of re-experiencing the trauma while answering must also be considered. Therefore, these scales should be used appropriately by experts so that proper support can be provided to such children and are not suitable for screening by school teachers. Other screening instruments include the Pediatric Hurt-Insult-Threaten-Scream-Sex (PedHITTSS) [14], and Psychosocial Assessment Tool (PAT) [15], which allow for screening of children’s traumatic experiences through caregivers’ assessment. However, as it is often the caregivers who perpetrate the maltreatment, it is difficult to detect maltreatment objectively through their assessment. In addition, many caregivers refuse to admit to the abuse, even when notified and presented with objective evidence. Consequently, the caregivers’ assessment may not be appropriate to provide support for such children and parents. For early detection of maltreatment, developing a scale that can be used for objective assessment by third parties in schools and other institutional settings is necessary.

The Attachment Disorder Assessment Scale-Revised (ADAS-R) is used in clinical attachment treatment in Australia for attachment disorder [16]. This scale includes various cultural items but cannot assess dissociative aspects. The Child Dissociative Checklist (CDC) is widely used in clinical practice in Japan to screen for dissociative symptoms in children [17]. However, it can assess dissociation but not attachment.

In this study, we aimed to develop a scale for use in a multidisciplinary, multi-agency setting for the early detection of maltreatment from the perspective of attachment and dissociation by combining and modifying the two existing scales (the ADAS-R and CDC). The resulting scale, the rating scale for maladaptive symptoms due to maltreatment (RS-MSM), aims to identify children with maltreatment symptoms from a large population. This scale can be used to detect maltreatment symptoms through the assessment of the behavior of children who have experienced maltreatment by third parties involved in the children’s daily lives rather than through the children’s own responses to their maltreatment experiences. This approach is meant to avoid causing psychological harm to the maltreated children by forcing them to recall traumatic experiences.

The RS-MSM may enable us to offer appropriate support to children who have been maltreated at an early stage. With earlier detection of maltreatment and referral to medical and welfare services, trained medical professionals would be able to conduct more detailed assessments on traumas including semi-structured interviews, with consideration for psychological invasiveness. Such involvement of the professionals could lead to earlier environmental adjustments and psychotherapy for children experiencing maltreatment.

## Materials and methods

The protocol of this study was approved by the Ethics Committee of the Graduate School of Human Sciences, Osaka University. It was conducted in accordance with the Declaration of Helsinki for experiments involving humans. A cross-sectional survey was conducted in schools in Osaka and Iwate prefectures, and orphanages in Iwate prefecture and Tokyo in Japan.

The survey consisted of two parts: 1) selecting questionnaire items and deciding the cut-off score, and 2) verifying the factor structure, reliability, and validity of the developed scale.

### Participants

The participants were categorized into two groups: maltreated group and the control group. Survey 1 included 60 and 154 children from the maltreated and control groups, respectively, whose care staff or homeroom teachers responded to all 40 questions.

Survey 1 included 60 elementary school students who lived in orphanages in Japan (Iwate prefecture and Tokyo) due to maltreatment (maltreated group: 30 boys and 30 girls, mean age 9.18 years, standard deviation [SD] 1.69, range 6–12 years). In addition, 154 elementary school children attending local public elementary schools in Japan (Osaka prefecture) with no confirmed history of maltreatment (control group: 70 boys, 73 girls, 11 with gender not stated, mean age 9.71 years, SD 1.69, range 6–12 years) were included in the analysis. Five children who had participated in the survey but turned out to have experienced maltreatment before analysis were excluded from the analysis.

In Survey 2, the maltreated group consisted of 39 elementary school children from the same orphanages as in Survey 1 (19 boys and 20 girls, mean age 9.06 years, SD 1.71, range 6–12 years). The control group consisted of 186 children from public elementary schools in Iwate and Osaka prefectures (70 boys, 78 girls, and 38 with gender not stated, mean age 9 years, SD 1.74; range 6–12 years). The flow chart of participants selection and participant characteristics for both surveys were shown in S1 Fig and S1 Table, respectively.

### Procedure

Survey 1 and Survey 2 were conducted from June to December 2020 and May to November 2021, respectively. In Survey 1, care staff at orphanages, who agreed to participate and gave written consent, were asked to rate children’s daily behavior using the 40 items for the maltreated group. Similarly, for the control group, homeroom teachers at public elementary schools, who gave written consent to participate, were asked to randomly select children with no confirmed history of abuse and assess their daily behavior. For accurate comparisons, it was desirable to use ratings by schoolteachers for both the groups. However, in Japan, due to the provisions of the child abuse prevention law and those of the criminal code on confidentiality and disclosure of secrets, questioning schoolteachers for specific evaluations of abused children was not possible. Thus, the orphanage staff rated the children in the maltreated group. School teachers rated those in the control group. Survey 2 was conducted using the rating scale as in Survey 1 for maladaptive symptoms due to maltreatment (RS-MSM) consisting of 20 items finalized through the analysis of Survey 1 results.

### Measures

We developed a scale to detect maltreatment in terms of attachment and dissociation.

An item pool of 40 questions was created to form a scale that screens for attachment and dissociative disorder symptoms. To screen for attachment disorder symptoms, 17 items measurable in the Japanese school setting were selected from the ADAS-R [16]. While the ADAS-R is a highly useful questionnaires, three additional items which are not included in the ADAS-R but often used to detect maltreatment in children via observational assessment of their behavior in clinical or school settings were added as follows: “Situational control (changing attitudes depending on people and situations),” “Hyper-vigilance (showing excessive caution or freezing up),” and “Trying behavior (behavior that tests people’s acceptance limits).” (S2 Table). The item “Situational control (changing attitudes depending on people and situations),” is an item that checks for the presence or absence of this behavior, as children who are maltreated can drastically change their attitudes from person to person [18, 19]. “Hyper-vigilance (showing excessive caution or freezing up),” is an item that assesses the presence or absence of excessive alertness or freezing up [20], and “Trying behavior (behavior that tests limits of acceptance by others).” is an item that checks for the presence of anxiety of abandonment, and the desire for social approval [21, 22]. These items were added to increase the accuracy of screening for children with overlapping attachment disorder and dissociative disorders, who would need intensive care and support at schools and in their daily lives.

Finally, 20 items were selected for attachment disorder. The CDC scale [17], consisting of 20 items, was used to screen for dissociative symptoms. Thus, a pool of 40 items was created (S2 Table) and items were answered on a 3-point Likert scale.

### Data analyses

In Survey 1, item response theory (IRT) and exploratory factor analysis (EFA) were used to select appropriate items for the scale. A graded response model [23] of the IRT was used to examine the item information functions (IIF) [24]. Item information is a function of latent trait (in this study, the level of maltreatment) measured by the responses to the items. The items with little information across the latent trait levels were excluded. In the EFA, the number of factors was determined by the Kaiser–Guttman criterion (Eigenvalue > 1) [25, 26] and Horn’s parallel analysis methods. Geomin rotation was performed, and items with factor loadings less than 0.4 and cross loadings were excluded [27]. Model fit was evaluated using the following goodness-of-fit indices: root mean square error of approximation (RMSEA) < 0.08 [28], comparative fit index (CFI)> 0.9, Tucker-Lewis Index (TLI)> 0.9 [29], standardized root mean square residual (SRMR) < 0.05 [30]. Cronbach’s alpha and composite reliability (CR) [31] were used to evaluate reliability. Cronbach’s alpha and CR were set at 0.70 or higher [28] as the standard values [32]. The average variance extracted (AVE) was used to assess convergent validity, with a criterion value of 0.50 or higher [33]. Receiver operating characteristic (ROC) curves were plotted for all participants to determine the cut-off values, with sensitivity and specificity on the x and y-axes, respectively, and area-under-curve (AUC) was calculated. The cut-off value was determined by considering the balance between sensitivity and specificity. P-value < 0.05 was set as the level of statistical significance.

In Survey 2, confirmatory factor analysis (CFA) was conducted to verify the factor structure, reliability, and validity of the scale. ROC analysis was performed, and CR, Cronbach’s alpha, and AVE were calculated.

Statistical analyses were conducted using Stata®IC ver. 16.1 (StataCorp LLC, College Station, TX, USA), Mplus ver. 8.5 (Muthen & Muthen, LA, CA, USA), and Microsoft Excel 2019 (Microsoft, Redmond, WA, USA).

## Results

**Fig 1:**
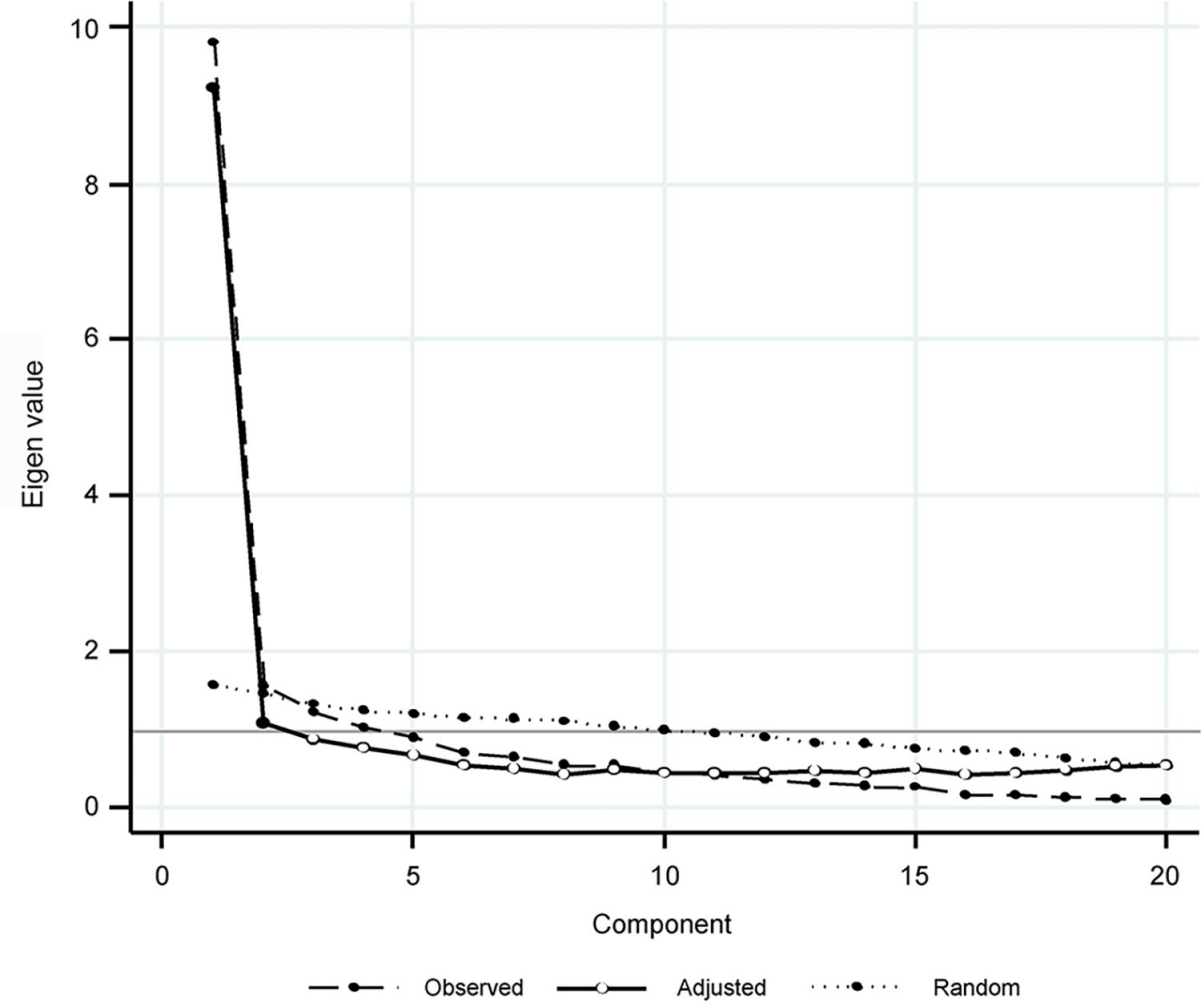
Survey 1 results of parallel analysis scree plot showing the first 10 factors.

The descriptive characteristics of the participants in Surveys 1 and 2 are depicted in S1 Table. No significant differences in age or gender between the maltreated and control groups were observed. On examining the item information functions, ten items that had little information across the latent trait levels were excluded from the pool of 40 items. Then, EFA was performed on the remaining 30 items. Two factors with Eigen values greater than 1.0 by parallel analysis were obtained (Fig 1). Ten items with factor loadings of 0.4 or less after the geomin rotation were excluded. The final scale consisted of 20 items. Of these, 13 items were derived from the attachment scale and 7 from the dissociation scale (S3 Table). The goodness-of-fit indices of the two-factor model were as follows: RMSEA = 0.041, CFI = 0.993, TLI = 0.991, and SRMR = 0.071. Although SRMR was slightly above 0.05, the other indices were highly compatible. The first factor had 12 items with factor loadings ranging from 0.51 to 0.93 and Cronbach’s alpha of 0.90; CR, 0.93; and AVE, 0.67 (S4 Table). The second factor consisted of 8 items with factor loadings ranging from 0.56 to 1.0. Cronbach’s alpha was 0.91; CR, 0.928; and AVE, 0.626. Cronbach’s alpha for the entire scale was 0.94. The first factor was related to social/interpersonal problems and dissociation, and the second was related to delinquent/aggressive behaviors (S3 Table).

**Fig 2:**
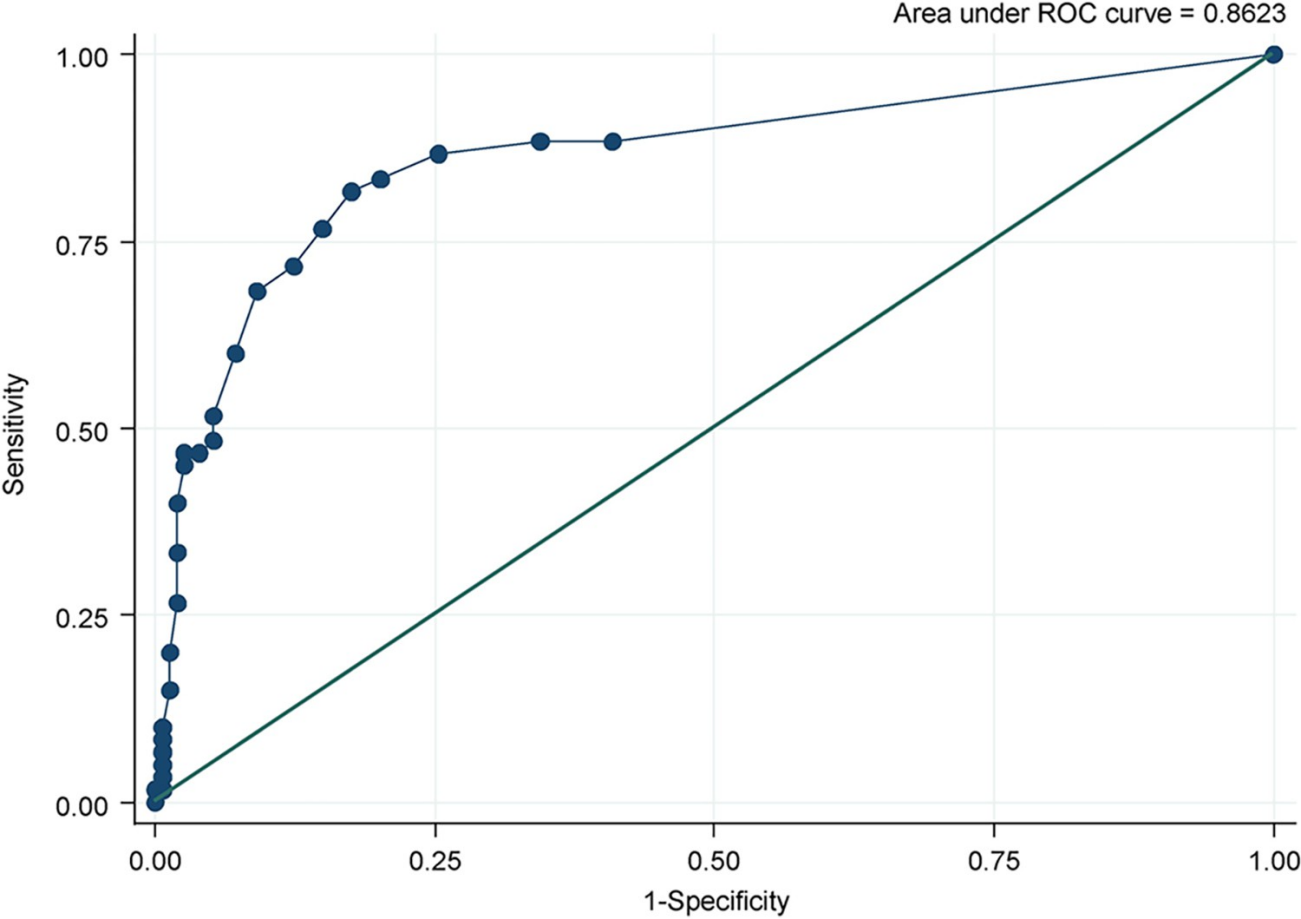
Receiver operating characteristics curve in Survey 1.

ROC curve yielded an AUC value of 0.86 (Fig 2). On examining the cut-off values of the scale from the ROC curve, the values were set at two levels for higher accuracy: one for screening the general population and the other for distinguishing the clinical population. The cut-offs were set at 2 (sensitivity 88.3 and specificity 65.5) and 5 (sensitivity 81.7% and specificity 82.5%).

Using the data obtained in Survey 2, two-factor CFA was conducted on the RS-MSM developed in Survey 1 (S5 Table). The goodness-of-fit indices were RMSEA = 0.044, CFI = 0.858, TLI = 0.840, and SRMR = 0.069. A sufficient model fit except for RMSEA was not obtained. On examination of the item-specific factor loadings, two items in the first factor were found to have factor loadings below 0.3.

The CR values for the first and second factors were 0.887 and 0.906, respectively, and both were high. The AVE value was 0.506 for the first factor and 0.557 for the second factor, and both were above the norm. Cronbach’s alpha for the entire scale was 0.93, which confirmed the reliability and convergent validity of the scale.

**Fig 3:**
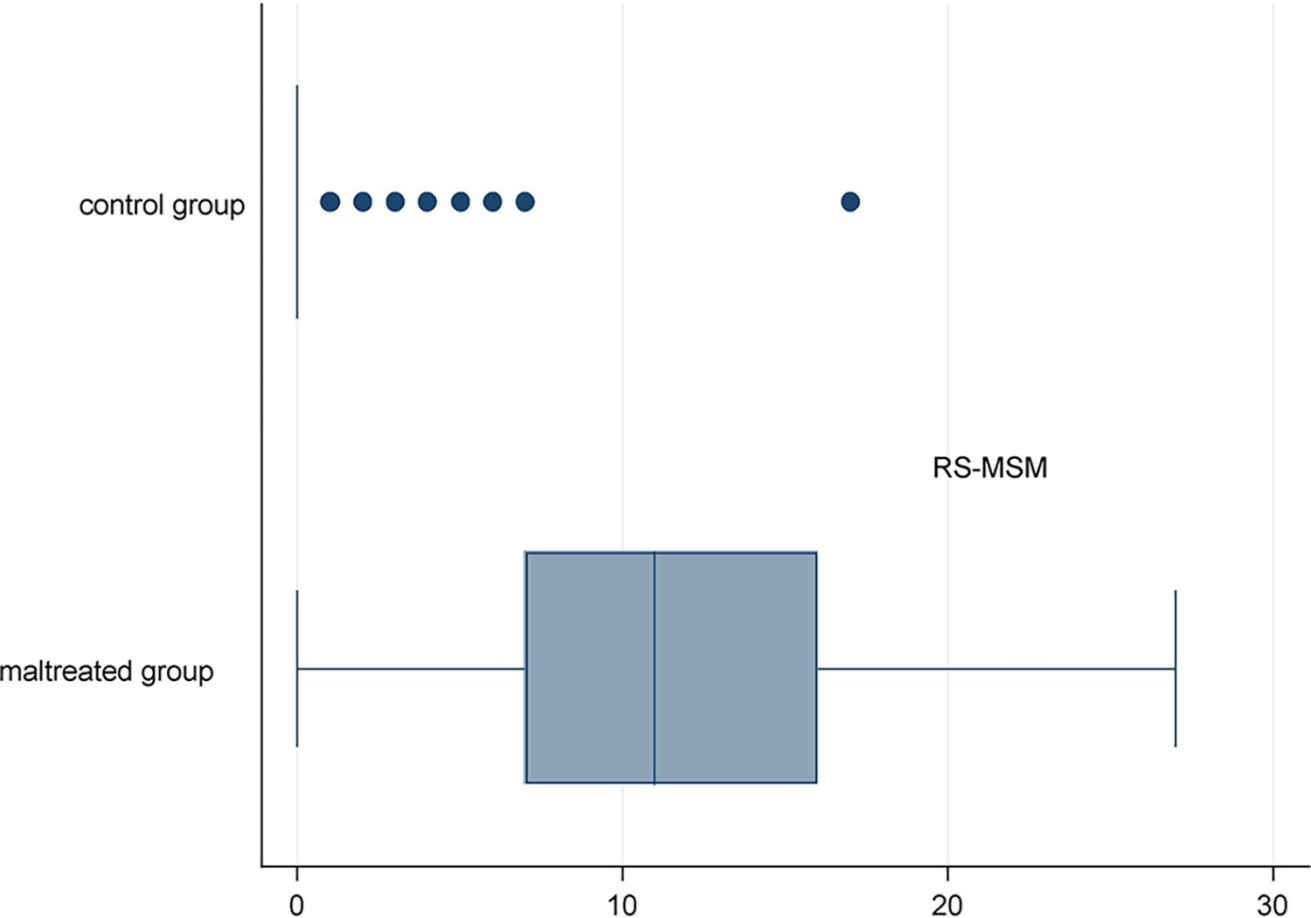
RS-MSM score distributions in the maltreatment group and the control group in Survey 2.

**Fig 4:**
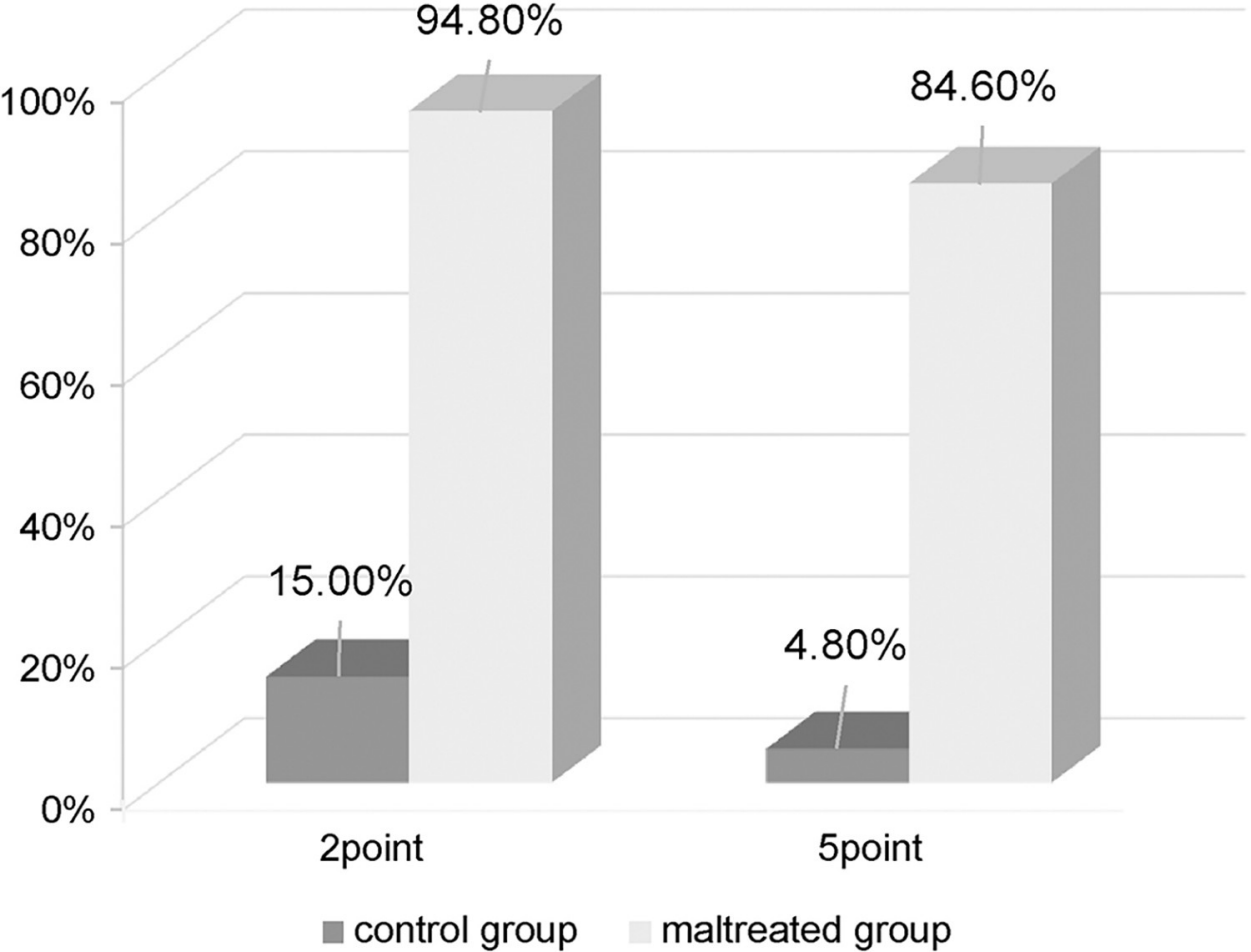
Percentage of participants with RS-MSM scores above cut-off.

Besides the ROC analysis, the cut-off values established in Survey 1 were examined on the sample of Survey 2. ROC analysis showed a sufficiently high AUC (0.96) [34, 35]. The comparative results of the total scores between the two groups are exhibited in Fig 3. When the cut-off was set at 2, the percentage of children above the cut-off was 94.9% (sensitivity) in the maltreated group and 14.5% in the control group (specificity = 85.5%); when the cut-off was set at 5, the percentage of children above the cut-off was 84.6% (sensitivity) in the maltreated group and 4.8% in the control group (specificity = 95.2%) (Fig 4).

## Discussion

In this study, a screening scale for symptoms caused by maltreatment through “evaluation of others” was developed, and its reliability and validity were confirmed. The resulting tool, the RS-MSM, can be used to screen children for maltreatment in schools and other settings and avoids children having to recall their painful experiences. It also allows schoolteachers and others involved in the children’s daily lives to objectively evaluate their behaviors, as opposed to the maltreating caregivers themselves.

Two of the three items added to those from the ADAS-R and CDC items were included in the RS-MSM. These two items measure situational control and trying behavior, which are often used to screen for symptoms of maltreatment in clinical settings, and were adopted as delinquent/aggressive behavior factors as a result of factor analysis. CFA showed that both approximate values, standard errors and *p*-values were highly correlated to maltreatment symptoms. This simple 20-item scale can screen for the presence of maltreatment based on children’s behavior observed in everyday situations, thereby connecting the children and their caregivers to appropriate levels of support. In addition, after carefully focusing on the child’s family environment with reference to the results of this scale, the teachers and the caregiver staff can deepen their understanding of the child’s problematic behaviors and devise appropriate ways to address these in an efficient manner. This improved understanding will enable the caregivers of a child who has been maltreated to provide support with shared purposes and goals, which may reduce inappropriate responses of the child in clinical situations.

In Survey 1, the children in the control group presented with more maltreatment-associated symptoms than in Survey 2. This difference could be attributed to the time Survey 1 was conducted, which coincided with the first wave of COVID-19 in Japan when school closures occurred, leading to drastic changes in the social and daily lives of children. In a survey on lifestyle changes, stress symptoms and their frequencies, and mental health of children during COVID-19 in Japan, it was shown that more than 70% of children had severe stress, such as “I hurt myself or my family” and “I get irritated easily” [36]. Such changes in the mental state of children without any history of maltreatment may have affected the results of Survey 1. Considering this, the effect of stress symptoms in the control group should be further explored by using the RS-MSM without COVID-19 related restrictions in the future.

We developed a questionnaire for screening maltreatment-associated symptoms in children by focusing on attachment and dissociation disorders. In the two-factor scale, the first factor was *social/interpersonal problems and dissociation*. The second factor was *delinquent/aggressive behaviors*. These did not correspond to simple “attachment disorder” and “dissociation.” The “social/ interpersonal problems and dissociation” factor consisted of items that represented maladaptive behavioral symptoms due to attachment disorder, such as feeling anxious, telling lies, and lack of emotional empathy. This factor seems to be consistent with the disorganized attachment style (Type D) in the Strange Situation Procedure (SSP) [37]. Type D attachment style is characterized by a state of conflict when reunited with the caregiver. Children with Type D attachment style often show contradictory behavior of simultaneous avoidance and approach and a friendly attitude toward new acquaintances. This attachment style crosses over the other attachment types: A (anxious-avoidant), B (stable), and C (resistant ambivalent/anxious). Children with Type D attachment styles are at a higher risk of developing dissociative disorders later in life [38]. A previous study showed its developmental trajectory, which starts with attachment problems, worsening dissociative symptoms with age, finally leading to aggression and multiple personalities [39]. A study examining the coexistence of maltreatment and Type D attachment style reported an incidence of Type D attachment in 82% and 17% of the population who were abused and those with no confirmed history of abuse, respectively [40]. As our focus while developing the RS-MSM was on attachment and dissociation, the items in this scale probably match the characteristics of Type D attachment. Numerous previous studies have shown that the prognosis of Type D attachment is poor [38, 39, 41]. Hence, it is essential to timely screen for attachment and dissociation issues, which are core symptoms of Type D attachment to provide proper treatment and support to the affected children.

Items for dissociative symptoms, such as trauma-induced amnesia and severe dissociative identity disorder, were included in the first factor. The second factor, “delinquent/aggressive behavior,” included items for high-risk and severe maladaptive symptoms, such as aggression towards self and others, and difficulties in controlling relationships with others. These are a part of the diagnostic criteria for complex PTSD, including maintaining relationships and proper distancing with others and difficulties in controlling emotions [9]. Both factors are important to confirm maladaptive behavior in children with maltreatment experiences.

In this study, we determined two cut-off scores. The cut-off score to identify maltreatment was set at 2 when screening the general population, considering the importance of not missing children who were maltreated. However, due to low specificity, using this cutoff may include those who have behavioral problems without maltreatment experiences. Applying the cutoff of 2, in a class of 35 students in Japan would have approximately five maltreatment-suspected students in a class, and it would be difficult for school counselors and social workers to immediately and intensively attend to all these students given the present school situation. Thus, for the children with a scores of 2–4, careful observation following the approach of trauma-informed care and gathering information regarding the home environment and other aspects should be conducted to further evaluate maltreatment presence or absence in more detail. Assessment of the child’s home environment is warranted even when the RS-MSM score is 2–4 because some items, especially those for the second factor, indicate severe symptoms.

Additionally, we also set the cut-off to 5 for the population with clinical symptoms to ensure children and their caregivers were promptly connected to appropriate support systems. The higher cutoff value of 5, which had a higher specificity, allowed for the identification of children experiencing maltreatment who needed urgent and specific supports. Setting these two cutoffs will enable schoolteachers, counselors, and social workers to take appropriate action that can be offered given the school’s practical situation.

The RS-MSM was found to have sufficient validity and reliability. Symptoms associated with maltreatment were identified in more than 80% of the children in the maltreated group, suggesting the need for psychological care. A safe environment is needed not only in the orphanages but also in the schools. The challenge is how schools can create safe places and provide appropriate support to children with identified maltreatment symptoms in both maltreated and control groups.

The teachers and staff need to be trained by school counselors, school social workers, and school psychology and social work professionals for providing appropriate response to the identified children with symptoms of attachment disorder and dissociation, so that they can be involved from the perspective of trauma-informed care. Effective support through cooperation among schools, specialized welfare institutions, and medical institutions may be necessary to solve problems at home, especially for children with severe symptoms such as dissociative symptoms and self-injury or harming others, after careful inspection of the actual situation. The RS-MSM developed in this study could enable schoolteachers, school supporters, school social workers, and other adults who support children with maladaptive behavior problems assess children based on their daily symptoms and identify whether there is existence of maltreatment. Consequently, it is expected to help identify children who need psychological and developmental support and link them to necessary medical and welfare support.

Sometimes it is difficult to distinguish between maltreatment and neurodevelopmental disorders when evaluated using screening scales for ASD and ADHD. It has also been found that there are differences between the neurobiological bases of ADHD-like behavior in children with childhood experiences of deprivation, and that of the developmental disorder of ADHD, in children without deprivation experiences [42]. The RS-MSM can distinguish between the responses to maltreatment but cannot tell the existence of neurodevelopmental characteristics such as ASD or ADHD. Future research should combine the RS-MSM with screening scales for developmental disorders to find ways to detect whether a child, who has been maltreated, has preexisting developmental disorders.

### Limitations

There are certain limitations to this study. First, in the CFA in Survey 2, the required goodness-of-fit indices for the analysis were not attained and the items in the scale had low factor loadings. This result was likely due to the smaller number of participants in the maltreated group and higher in the control group compared to Survey 1, which changed the probability of responses. Second, the sample size of maltreated children was small considering the heterogeneity of maltreatment. However, similar reliability and validity were found in two different sets of samples in this study, which suggest the usefulness of the scale. Third, the consistency of ratings among the respondents could not be assessed. This study conducted evaluations by care staff in the orphanages for the maltreated group and homeroom teachers for the control group due to ethical constraints. As stated in the Methods section, we could not pick up specific children, who have been abused, to be rated by homeroom teachers in schools due to the privacy protection under the child abuse prevention law in Japan. Nevertheless, in Survey 1, several homeroom teachers answered the questionnaire for five children without knowing their histories of abuse, or whose abuse histories were identified after the survey. These data were excluded from the analysis; however, the results showed that all five scored above the RS-MSM cut-off score of 5 (S6 Table). This suggests that the RS-MSM shows similar results even if the raters have different occupational backgrounds, even considering the small samples size. In addition, although the teachers and orphanage staff had different occupational backgrounds, training materials for both of them have been provided by the Ministry of Education, Culture, Sports, Science and Technology and the Ministry of Health, Labor and Welfare, as well as training programs on managing children with an abusive or attachment disorder background by doctors and specialists in Japan.

The RS-MSM scale was developed as an assessment tool to help teachers and support workers assess the presence or absence of maltreatment symptoms in children whom they work with so they can be linked to specialist agencies such as health and social services. In the future, to extend the research and confirm the current study’s results, different types of respondents will be included, such as those from medical and various welfare agencies.

## Conclusion

We developed the RS-MSM, a scale that enables screening for maladaptive symptoms in children who have been maltreated from dissociation and attachment perspectives. By combining the existing scales on attachment and dissociation, this developed scale which can assess maltreatment symptoms will enable school teachers, social workers and others who support children with maladaptive behavioral problems to identify children experiencing maltreatment. The RS-MSM consisted of two factors for which sufficient reliability and convergent validity were established.

The RS-MSM developed in this study will help identify children who need support for maladaptive behavioral problems and connect them to get adequate support.

## Supporting information

S1 Fig Participant flowchart of Survey1 and 2. (DOCX)

S1 Table Demographic characteristics of the participants. (DOCX)

S2 Table Questionnaire items used in Survey 1. (DOCX)

S3 Table The rating scale for maladaptive symptoms due to maltreatment (RS-MSM) Questionnaire items. (DOCX)

S4 Table Rotated factor matrix for attachment and dissociation scale items in Survey 1. (DOCX)

S5 Table Confirmatory factor analysis of RS-MSM questionnaire items in Survey 2. (DOCX)

S6 Table RS-MSM scores by teachers of five children experiencing maltreatment. (DOCX)

S7 Table Survey 1 raw data. (XLSX)

S8 Table Survey 2 raw data. (XLSX)
