# Supplementary figures and images for: Development of a rating scale for maladaptive symptoms by maltreatment: Perspectives of attachment and dissociation
Source: PLoS One. 2024 Feb 14;19(2):e0298214. doi: 10.1371/journal.pone.0298214 (PMC10866495; doi:10.1371/journal.pone.0298214)

**S1 Figure. Participant flowchart of Surveys 1 and 2**


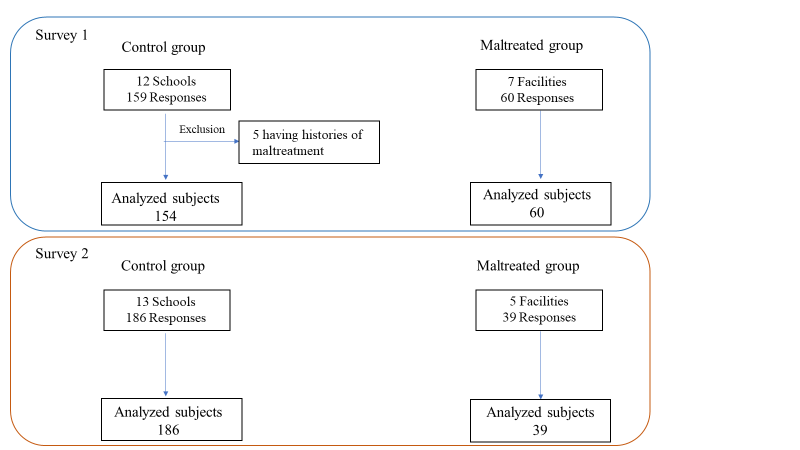

Supplement: S1 Fig — (DOCX) [file pone.0298214.s001.docx]
