# Supplementary material for: Development of a rating scale for maladaptive symptoms by maltreatment: Perspectives of attachment and dissociation
Source: PLoS One. 2024 Feb 14;19(2):e0298214. doi: 10.1371/journal.pone.0298214 (PMC10866495; doi:10.1371/journal.pone.0298214)
Supplement: S1 Table — (DOCX) [file pone.0298214.s002.docx]

**S2 Table. Demographic characteristics of the participants.**

| **Survey 1** |  | **Maltreated group** | **Control group** |
| --- | --- | --- | --- |
| Number of children |  | 60 | 154 |
| Gender, N (%) | Male | 30 (50) | 70 (45.5) |
|  | Female | 30 (50) | 73 (47.4) |
|  | Unknown | 0 (0) | 11 (7.1) |
| Age (year) | Mean | 9.18 | 9.71 |
|  | SD | 1.62 | 1.69 |
|  | Range | 6-12 | 6-12 |
| **Survey 2** |  |  |  |
| Number of children |  | 39 | 186 |
| Gender, N (%) | Male | 19 | 70 |
|  | Female | 20 | 78 |
|  | Unknown | 0 | 38 |
| Age (year) | Mean | 9.06 | 9 |
|  | SD | 1.71 | 1.74 |
|  | Range | 6-12 | 6-12 |

*Note.* SD, standard deviation.
