# Supplementary material for: Development of a rating scale for maladaptive symptoms by maltreatment: Perspectives of attachment and dissociation
Source: PLoS One. 2024 Feb 14;19(2):e0298214. doi: 10.1371/journal.pone.0298214 (PMC10866495; doi:10.1371/journal.pone.0298214)
Supplement: S2 Table — (DOCX) [file pone.0298214.s003.docx]

**S3 Table: Questionnaire items used in Survey 1**

*Selected items from the Attachment Disorder Assessment Scale-Revised (ADAS-R)*

17 items

1. The child indiscriminately approaches a stranger.

2. The child lacks internalized moral reasoning and lacks a conscience.

3. They frequently lie with or without apparent reason.

4. The child does not seem to understand the meaning of remorse or giving a sincere apology.

5. The child possesses a serious lack of empathy for others.

6. The child steals or takes what does not belong to them.

7. They make excessive demands and are anxious, angry, or clingy when demands are unmet.

8. The child attacks peers and/or threatens adults.

9. They tend to be self-centered.

10. The child exhibits self-destructive behaviors (attempting self-harm).

11. This child is hurtful to animals.

12. This child does not seem to understand the meaning of remorse or giving a sincere apology.

13. They have trouble making eye contact.

14. No matter how much time I spend with this child, I do not believe I can get any closer to them emotionally.

15. This child responds to friendly overtures with avoidance or isolating behavior.

16. This child resists being comforted.

17. The child attempts to be in control of everything (they may speak ill of others and give orders).

*Item 17 adds expressive examples of children's behavior seen in clinical practice in Japan.

*Additional attachment-related questions*

3 items

1. The child can change their attitude and appear very differently depending on the people and teachers involved.

2. The child experiences situations where they are overly alert or freeze up.

3. The child exhibits behavior that tests how far people will accept him/her.

*Child Dissociative Checklist (CDC) (Version 3 by Frank W. Putnam, MD)*

20 items

1. The child does not remember or denies traumatic or painful experiences that are known to have occurred.

2. The child sometimes goes into a daze or trance-like state or often appears "spaced out." Teachers may report that they "daydream" frequently in school.

3. The child shows rapid personality changes. They may go from being shy to being outgoing, from feminine to masculine, from timid to aggressive.

4. The child is unusually forgetful or confused about things they should know, e.g., they may forget the names of friends, teachers, or other important people, loses possessions, or gets easily lost.

5. The child has a very poor sense of time. They lose track of time, may think it is morning when it is actually afternoon, get confused about what day it is, or become confused about when something happened.

6. The child shows marked day-to-day or even hour-to-hour variations in their skills, knowledge, food preferences, and athletic abilities, e.g., changes in handwriting, memory for previously learned information such as multiplication tables, spelling, use of tools, or artistic ability.

7. The child shows rapid regressions in age-level behavior, e.g., a twelve-year-old starts to baby talk, sucks their thumb, or draws like a four-year-old.

8. The child has difficulty learning from experience, e.g., explanations, normal discipline, or punishment do not change their behavior.

9. The child continues to lie or deny misbehavior despite obvious evidence.

10. The child refers to themselves in the third person (e.g., as them) when talking about self or sometimes insists on being called by a different name. They may also claim that something that they did actually happened to another person.

11. The child expresses rapidly changing physical complaints such as headache or upset stomach. For example, they may complain of a headache one minute and seem to forget about it the next.

12. The child is unusually sexually precocious and may attempt age-inappropriate sexual behavior with other children or adults.

13. The child suffers from unexplained injuries or may even deliberately self-injure at times.

14. The child reports hearing voices. The voices may be friendly or angry and may come from "imaginary companions" or sound like the voices of parents, friends, or teachers.

15. The child has a vivid imaginary companion or companions. The child may insist that the imaginary companion(s) is responsible for things that they have done.

16. The child has intense outbursts of anger, often without apparent cause, and may display unusual physical strength during these episodes.

17. The child sleepwalks frequently.

18. The child has unusual nighttime experiences, e.g., may report seeing "ghosts" or that things happen at night that they can't account for (e.g., broken toys, unexplained injuries)

19. The child frequently talks to themself and may sometimes use a different voice or argue with themselves.

20. The child has two or more distinct and separate personalities that take control of the child's behavior.
