# Supplementary material for: Development of a rating scale for maladaptive symptoms by maltreatment: Perspectives of attachment and dissociation
Source: PLoS One. 2024 Feb 14;19(2):e0298214. doi: 10.1371/journal.pone.0298214 (PMC10866495; doi:10.1371/journal.pone.0298214)
Supplement: S3 Table — (DOCX) [file pone.0298214.s004.docx]

**S4 Table The** **rating scale for maladaptive symptoms due to maltreatment (RS-MSM)　questionnaire items**

**<Social/Interpersonal problems and dissociation>**

1. The child indiscriminately approaches a stranger. (A)

2. The child lacks internalized moral reasoning and lacks a conscience. (A)

3. They frequently lie, with or without apparent reason. (A)

4. The child does not seem to understand the meaning of remorse or giving a sincere apology. (A)

5. The child exhibits a serious lack of empathy for others. (A)

6. The child shows rapid personality changes. They may go from being shy to being outgoing, from feminine to masculine, from timid to aggressive. (D)

7. The child is unusually forgetful or confused about things they should know, e.g., they may forget the names of friends, teachers, or other important people, loses possessions, or gets easily lost. (D)

8. The child has a very poor sense of time. They lose track of time, may think it is morning when it is actually afternoon, get confused about what day it is, or become confused about when something happened. (D)

9. The child shows rapid regressions in age-level behavior, e.g., a twelve-year-old starts to use baby talk, sucks their thumb, or draws like a four-year-old. (D)

10. The child has difficulty learning from experience, e.g., explanations, normal discipline, or punishments do not change their behavior. (D)

11. The child exhibits rapidly changing physical complaints such as headache or upset stomach. For example, they may complain of a headache one minute and seem to forget about it the next. (D)

12. The child has a vivid imaginary companion or companions. The child may insist that the imaginary companion(s) is responsible for things that they have done. (D)

**<Delinquent/Aggressive behaviors>**

1. The child steals or takes what does not belong to them. (A)

2. They make excessive demands and are anxious, angry, or clingy when demands are unmet. (A)

3. The child attacks peers and/or threatens adults. (A)

4. They tend to be self-centered. (A)

5. The child exhibits self-destructive behaviors (attempting self-harm). (A)

6. The child exhibits behavior that tests how far people will accept them. (A)

7. The child attempts to control everything (they may speak ill of others and give orders). (A)

8. They can change their attitudes and appear very differently depending on the people and teachers involved. (A)

*Note.* “A” stands for items from the 20 questions regarding attachment and “D” for items from the Child Dissociative Checklist*.*
