# Supplementary material for: Development of a rating scale for maladaptive symptoms by maltreatment: Perspectives of attachment and dissociation
Source: PLoS One. 2024 Feb 14;19(2):e0298214. doi: 10.1371/journal.pone.0298214 (PMC10866495; doi:10.1371/journal.pone.0298214)
Supplement: S4 Table — (DOCX) [file pone.0298214.s005.docx]

**S5 Table. Rotated factor matrix for attachment and dissociation scale items in Survey 1.**

|  | **Factor 1** | **Factor 2** |
| --- | --- | --- |
| Attachment1 | 0.513＊ | 0.345 |
| Attachment2 | 0.697＊ | 0.316 |
| Attachment3 | 0.632＊ | 0.276 |
| Attachment17 | 0.744＊ | 0.267 |
| Attachment18 | 0.732＊ | 0.256 |
| Dissociation3 | 0.678＊ | 0.205 |
| Dissociation4 | 0.856＊ | -0.025 |
| Dissociation5 | 0.933＊ | -0.165 |
| Dissociation7 | 0.828＊ | 0.001 |
| Dissociation8 | 0.717＊ | 0.265 |
| Dissociation11 | 0.875＊ | -0.057 |
| Dissociation15 | 0.649＊ | 0.038 |
| Attachment5 | 0.234 | 0.572＊ |
| Attachment6 | 0.267 | 0.758＊ |
| Attachment7 | -0.036 | 1.004＊ |
| Attachment8 | 0.146 | 0.839＊ |
| Attachment10 | 0.005 | 0.897＊ |
| Attachment11 | 0.244 | 0.708＊ |
| Attachment13 | -0.014 | 0.860＊ |
| Attachment15 | 0.357 | 0.566＊ |
| **Inter-factor correlation** | |  |
| **Factor** | **1** | **2** |
| 1 | 1.000 | 0.710 |
| 2 | 0.710 | 1.000 |

*Note.* GEOMIN factor correlations/significant at the 5% level.

＊*p* < 0.05.
