# Supplementary material for: Development of a rating scale for maladaptive symptoms by maltreatment: Perspectives of attachment and dissociation
Source: PLoS One. 2024 Feb 14;19(2):e0298214. doi: 10.1371/journal.pone.0298214 (PMC10866495; doi:10.1371/journal.pone.0298214)
Supplement: S5 Table — (DOCX) [file pone.0298214.s006.docx]

**S6 Table. Confirmatory factor analysis of RS-MSM questionnaire items in Survey 2.**

| **Item** | **Est** | **SE** | **Est/SE** | **Two-tailed**  ***p*-value** |
| --- | --- | --- | --- | --- |
| Factor 1 |  |  |  |  |
| F1-1 | 0.653 | 0.057 | 11.422 | 0.000 |
| F1-2 | 0.842 | 0.030 | 28.358 | 0.000 |
| F1-3 | 0.834 | 0.042 | 19.646 | 0.000 |
| F1-4 | 0.835 | 0.051 | 16.472 | 0.000 |
| F1-5 | 0.776 | 0.081 | 9.621 | 0.000 |
| F1-6 | 0.621 | 0.087 | 7.106 | 0.000 |
| F1-7 | 0.517 | 0.093 | 5.578 | 0.000 |
| F1-8 | 0.246 | 0.083 | 2.980 | 0.003 |
| F1-9 | 0.520 | 0.099 | 5.232 | 0.000 |
| F1-10 | 0.806 | 0.048 | 16.872 | 0.000 |
| F1-11 | 0.457 | 0.089 | 5.158 | 0.000 |
| F1-12 | 0.297 | 0.103 | 2.878 | 0.004 |
| Factor 2 |  |  |  |  |
| F2-1 | 0.528 | 0.099 | 5.327 | 0.000 |
| F2-2 | 0.859 | 0.038 | 22.570 | 0.000 |
| F2-3 | 0.810 | 0.049 | 16.507 | 0.000 |
| F2-4 | 0.841 | 0.036 | 23.337 | 0.000 |
| F2-5 | 0.475 | 0.100 | 4.744 | 0.000 |
| F2-6 | 0.86 | 0.035 | 24.571 | 0.000 |
| F2-7 | 0.645 | 0.086 | 7.532 | 0.000 |
| F2-8 | 0.836 | 0.043 | 19.519 | 0.000 |

*Note.* Est, estimated value; SE, standard error.
