# Supplementary material for: Development of a rating scale for maladaptive symptoms by maltreatment: Perspectives of attachment and dissociation
Source: PLoS One. 2024 Feb 14;19(2):e0298214. doi: 10.1371/journal.pone.0298214 (PMC10866495; doi:10.1371/journal.pone.0298214)
Supplement: S6 Table — (DOCX) [file pone.0298214.s007.docx]

**S7 Table:** **RS-MSM scores by teachers of five children who were maltreated**

| **Mean value** | | **Calculated value** | |
| --- | --- | --- | --- |
| Mean | | 16.4 |  |
| Median |  | 15 |  |
| Minimum value | | 7 |  |
| Greatest value | | 32 |  |
